# Free radical derivatives formed from cyclooxygenase-catalyzed dihomo-γ-linolenic acid peroxidation can attenuate colon cancer cell growth and enhance 5-fluorouracil׳s cytotoxicity

**DOI:** 10.1016/j.redox.2014.01.022

**Published:** 2014-03-20

**Authors:** Yi Xu, Jin Qi, Xiaoyu Yang, Erxi Wu, Steven Y. Qian

**Affiliations:** a Department of Pharmaceutical Sciences, College of Pharmacy, Nursing and Allied Sciences, North Dakota State University, Fargo, ND 58108, USA; b Department of Complex Prescription of TCM, China Pharmaceutical University, Nanjing, Jiangsu 211198, China

**Keywords:** AA, arachidonic acid, ACN, acetonitrile, COX, cyclooxygenase, DGLA, dihomo-γ-linoleic acid, DHA, docosahexaenoic acid, D5D, delta-5 desaturase, EIC, extracted ion chromatogram, EPA, eicosapentaenoic acid, ESR, electron spin resonance, GC, gas chromatography, HEX, 1-hexanol, HOAc, glacial acetic acid, HPLC/LC, high performance liquid chromatography, HTA, heptanoic acid, MS, mass spectrometry, POBN, *α*-[4-pyridyl-1-oxide]-*N-tert*-butyl nitrone, PGs, prostaglandins, PI, propidium iodide, PUFA, polyunsaturated fatty acid, SPE, solid phase extraction, TBS, Tris buffered saline, TIC, total ion chromatogram, 5-FU, 5-Fluorouracil, 8-HOA, 8-hydroxyoctanoic acid, Cell cycle and apoptosis, Colon cancer cell line HCA-7 colony 29, COX-catalyzed PUFA peroxidation, DGLA׳s free radical derivatives, 5-Fluorouracil, LC/MS and ESR spin trapping

## Abstract

Dihomo-γ-linolenic acid (DGLA) and its downstream fatty acid arachidonic acid (AA) are both nutritionally important ω–6 polyunsaturated fatty acids (ω–6s). Evidence shows that, via COX-mediated peroxidation, DGLA and its metabolites (1-series prostaglandins) are associated with anti-tumor activity, while AA and its metabolites (2-series prostaglandins) could be tightly implicated in various cancer diseases. However, it still remains a mystery why DGLA and AA possess contrasting bioactivities. Our previous studies showed that DGLA could go through an exclusive C-8 oxygenation pathway during COX-catalyzed lipid peroxidation in addition to a C-15 oxygenation pathway shared by both DGLA and AA, and that the exclusive C-8 oxygenation could lead to the production of distinct DGLA׳s free radical derivatives that may be correlated with DGLA׳s anti-proliferation activity. In the present work, we further investigate the anti-cancer effect of DGLA׳s free radical derivatives and their associated molecular mechanisms. Our study shows that the exclusive DGLA׳s free radical derivatives from C-8 oxygenation lead to cell growth inhibition, cell cycle arrest and apoptosis in the human colon cancer cell line HCA-7 colony 29, probably by up-regulating the cancer suppressor p53 and the cell cycle inhibitor p27. In addition, these exclusive radical derivatives were also able to enhance the efficacy of 5-Fluorouracil (5-FU), a widely used chemo-drug for colon cancer. For the first time, we show how DGLA׳s radical pathway and metabolites are associated with DGLA׳s anti-cancer activities and able to sensitize colon cancer cells to chemo-drugs such as 5-FU. Our findings could be used to guide future development of a combined chemotherapy and dietary care strategy for colon cancer treatment.

## Highlight

• The free radical metabolites from DGLA peroxidation may possess anticancer activity.
• The DGLA׳s radical derivatives could induce cell cycle arrest in colon cancer cell.
• The DGLA׳s radical derivatives could induce apoptosis in colon cancer cell.
• The DGLA׳s radical derivatives could sensitize colon cancer cell to 5-fluororacil.

## Graphical abstract

**Figure:**
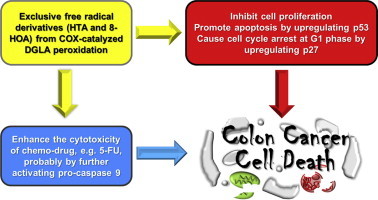

## Introduction

There are two major types of polyunsaturated fatty acids (PUFAs), namely, ω−6s and ω–3s. The ω–3s, such as eicosapentaenoic acid (EPA) and docosahexaenoic acid (DHA), are mainly found in seafood and are extensively studied for their beneficial effects, including improvement of cognitive ability and prevention of cancer and cardiovascular diseases [1–4]. The ω−6s, such as dihomo-γ-linolenic acid (DGLA) and arachidonic acid (AA), present in most plant oils, are more abundant in our daily diet than ω–3s. However, research on ω–6s׳ beneficial effects on human health has received much less attention. Upon catalysis of cyclooxygenase (COX), both DGLA and AA can undergo radical-mediated lipid peroxidation and produce prostaglandins: 1-series (PGs-1) and 2-series prostaglandins (PGs-2), respectively. It has been widely accepted that AA could be tightly implicated in inflammatory disorders and cancer development due to the formation of PGs-2 [5–10]. On the other hand, increasing evidence has suggested that DGLA, the precursor of AA (converted from DGLA by delta-5 desaturase, D5D), may represent an exceptional ω–6 by virtue of its anti-inflammatory and anti-cancer effects [11–17]. For instance, a dietary DGLA supplement helped to prevent atherosclerosis in ApoE-deficient mice; treatment with PGs-1 led to anti-neoplastic effects in B16-F10 murine melanoma cells; and DGLA treatment exerted direct cytotoxicity towards human cervical carcinoma cells (both drug-sensitive and resistant) [13,15,16]. Although evidence showed that DGLA׳s bioactivities were partially attributable to its COX-mediated metabolites, e.g., PGs-1 and/or free radicals, how DGLA could affect cancer cell growth still remains unclear.

COX-2, a key enzyme regulating PUFA peroxidation, is overexpressed in 80–90% of colon cancer cells and closely implicated in cancer development [18–20]. Under the catalysis of COX, both DGLA and its downstream PUFA (e.g., AA) can undergo free radical-mediated lipid peroxidation to produce a variety of free radical species as well as PGs. A number of research efforts have been focused on PUFAs׳ and PGs׳ diverse bioactivities; some of them suggested that PUFAs may regulate cell growth independently of PGs [5,11,12,15,21]. Although free radical-mediated activity from COX-catalyzed lipid peroxidation has been well accepted, the potential bioactivity of individual free radical intermediates has not been investigated previously due to the lack of appropriate methods to identify and characterize the free radical species.

**Scheme 1:**
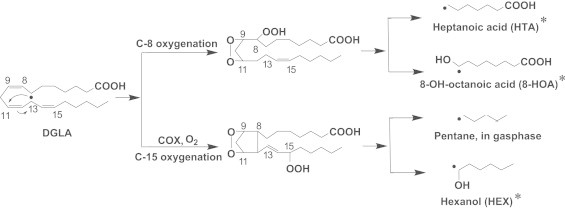
Proposed pathway and formation of radical derivatives in COX-catalyzed DGLA peroxidation. During the COX-catalyzed lipid peroxidation, DGLA can go through a unique C-8 oxygenation reaction pathway, in addition to a C-15 oxygenation pathway shared by both of DGLA and AA. The C-8 oxygenation produces two exclusive DGLA-derived free radical metabolites, ^•^C_7_H_13_O_2_ and ^•^C_8_H_15_O_3_, while the C15 pathway produces two common radical metabolites, ^•^C_6_H_13_O and ^•^C_5_H_11_. The asterisked products, heptanoic acid (HTA), 8-OH-octanoic acid (8-HOA) and 1-hexanol (HEX), are expected to form from free radicals by abstracting an H^•^ from the environment in the absence of the spin trapping agent POBN. The common free radical ^•^C_5_H_11_ produced via the C15 pathway is mainly present in the gas phase, thus was not tested in this study.

Using a novel HPLC/ESR/MS combined technique, our previous study successfully detected and identified major free radical metabolites formed from COX/DGLA and COX/AA peroxidation [22–26]. We showed that despite a common C-15 oxygenation pathway shared by both DGLA and AA (from the same structural moiety), there is a C-8 oxygenation pathway present only in COX/DGLA peroxidation (Scheme 1) [23,24]. This exclusive C-8 oxygenation leads to the production of two exclusive DGLA׳s free radical metabolites, ^•^C_7_H_13_O_2_ and ^•^C_8_H_15_O_3_ (observed as POBN-trapped adducts [23,24]), which are expected to form the corresponding radical derivatives heptanoic acid (HTA) and 8-hydroxyoctanoic acid (8-HOA), respectively (Scheme 1). In the present study, these radical derivatives along with 1-hexanol (HEX, the common radical derivative formed from both AA and DGLA) were directly used to test their potential anti-cancer activity and mechanisms in HCA-7 colony 29 cells, a human colon cancer cell line with high COX expression. Our results suggest that the exclusive DGLA׳s free radical derivatives (and therefore COX-catalyzed DGLA peroxidation) lead to cell growth inhibition, cell cycle arrest and apoptosis in colon cancer cells, probably by up-regulating the cancer suppressor p53 and cell cycle inhibitor p27.

The major obstacle for cancer chemotherapy has been drug resistance. For example, insensitivity to 5-Fluorouracil (5-FU), a commonly used chemotherapy drug in colon cancer treatment, has been reported in many colon cancer cell lines [27,28,31]. Various combination treatments of 5-FU with other chemo-drugs and agents have been tested in order to improve 5-FU׳s efficacy [29–32]. For example, the combined treatment of 5-FU with a COX inhibitor to regulate PUFA metabolism (especially to inhibit COX/AA metabolism to limit PGs-2 formation) has been shown to be a promising strategy for colon cancer therapy [31]. Here, we have proposed and demonstrated that the exclusive DGLA׳s radical derivatives, and therefore the radical reactions of COX-catalyzed DGLA peroxidation, actually sensitize HCA-7 colony 29 colon cancer cells to the chemotherapy drug 5-FU. Our research findings could be used to guide the development of a combined chemotherapy and dietary care strategy for colon cancer treatment by taking advantage of the commonly high level of COX in colon cancer cells and a more pervasive PUFA in the daily diet (ω−6 DGLA, instead of ω−3s).

## Methods and materials

### Cell line

The human colon cancer cell line HCA-7 colony 29 (European Collection of Cell Cultures, Salisbury, UK) was grown in Dulbecco׳s modified Eagle׳s medium high-glucose medium (Thermo Fisher Scientific, UT, USA) supplemented with 10% fetal bovine serum (Thermo Fisher Scientific, UT, USA) and cultured in an incubator containing a 95% humidified atmosphere with 5% CO_2_ at 37 °C. Cells were sub-cultured at a ratio of 1:5–1:8 after they had reached ~90% confluency.

### Detection of PUFAs and PGs from DGLA-treated cells

The PUFAs and PGs from HCA-7 colony 29 cells treated with DGLA were quantified via LC/MS analysis as described elsewhere [24]. Briefly, 2×10^6^ cells (in 4 mL cell culture medium) were seeded overnight in a 100-mm petri dish at 30–40% confluency and treated with DGLA (100 µM). At different time points, 2 mL of cell culture medium was collected and mixed with internal standards (AA-d_8_, DGLA-d_6_, PGE1-d_4_ and PGE2-d_9_). Then 0.45 mL of methanol and 0.55 mL water were added to make a total of 3 mL of 15% methanol solution. The mixture was vortexed for 1 min and set on ice for 30 min. After it was centrifuged for 15 min at 3000 rpm, the supernatant was collected and adjusted to pH 3.0, then subjected to solid phase extraction (SPE) using a reverse phase SPE cartridge (SampliQ Silica C18 ODS, Agilent, CA, USA). The extract was eluted with 2 mL ethyl acetate, and the elution dried under vacuum and reconstituted with 100 µL ethanol for LC/MS analysis.

The LC/MS system consisted of an Agilent 1200 series HPLC system and an Agilent 6300 LC/MSD SL ion trap mass system. LC separations were performed on a C18 column (Zorbax Eclipse-XDB, 4.6×75 mm, 3.5 μm) with an injection volume of 5 μL, flow rate at 0.8 mL/min and mobile phases of A: H_2_O-0.1% HOAc, and B: ACN-0.1% HOAc. Gradients were (1) 0–12 min (isocratic), 68% A and 32% B; (2) 12–14 min, 68–44% A and 32–56% B; (3) 14–28 min (isocratic), 44% A and 56% B; (4) 28–30 min, 44–14% A and 56–86% B; (5) 30–38 min, 14–4% A and 86–95% B; and (6) 38–44 min (isocratic), 5% A and 95% B. MS settings were as follows: electrospray ionization in negative mode; total ion current (TIC) chromatograms were performed in full mass scan mode (*m*/*z* 50 to *m*/*z* 600); nebulizer press, 15 psi; dry gas flow rate, 5 L/min; dry temperature, 325 °C; compound stability, 20%; number of scans, 50. The concentrations of PUFAs and PGs were quantified by comparing the ratios of the peak areas of the PUFAs and PGs to the internal standards.

### Detection of free radicals from DGLA-treated cells

The free radicals produced from HCA-7 colony 29 cells treated with DGLA were detected and quantified via LC/MS analysis along with spin trapping methods as described elsewhere [24]. Briefly, 2×10^6^ cells (in 3 mL phenol red free cell culture medium) were seeded overnight in a 100 mm petri dish at 30–40% confluency and treated with POBN (α-[4–pyridyl–1–oxide]-N-tert-butyl nitrone, 20 mM) and DGLA (100 µM) to start the peroxidation and spin-trapping reaction. At different time points, the cell culture medium and cell homogenate were collected and mixed with an equal volume of acetonitrile (ACN) to stop the reaction. Then the mixture was vortexed and centrifuged for 15 min at 3000 rpm. The supernatant was collected and subjected to SPE using a mixed-mode anion exchange SPE cartridge (Oasis MAX, Waters, MA, USA), followed by LC/MS analysis. Instead of detecting the ESR-active POBN-trapped free radical adduct, we actually measured the reduced form of radical adducts (e.g. hydroxylamines) since they are a more stable redox form and could accumulate during incubation [24].

The same LC/MS system was employed as used in the detection of PUFAs and PGs. LC separation was performed with the injection volume of 40 μL, flow rate at 0.8 mL/min and gradient of: (1) 0–5 min, 90–73% A and 10–27% B; (2) 5–25 min (isocratic), 73% A and 27% B; (3) 25–40 min, 73–30% A and 27–70% B; (4) 40–43 min, 30–5% A and 70 to 95% B; and (5) 43–50 min (isocratic), 5% A and 95% B. MS settings are as follows: electrospray ionization in positive mode; TIC was performed in full mass scan mode (*m/z* 50 to *m/z* 600); capillary voltage, −4500 V; nebulizer press, 20 psi; dry gas flow rate, 8 L/min; dry temperature, 60 °C; compound stability, 20%; and number of scans, 50. An extracted ion current chromatogram (EIC) was obtained to acquire the MS profile of the individual POBN trapped radical adduct. A deuterated spin trap (d_9_-POBN) was used as an internal standard for quantification as described elsewhere [22,24,33,34].

### Cell proliferation assay (MTS)

Cell proliferation after different treatments was assessed using CellTiter^®^ 96 Aqueous One Solution Reagent (Promega, Madison, WI, USA) according to the manufacturer׳s instructions. Briefly, the cells were seeded at 8000 cells (in 100 μL medium) per well into 96-well plates, incubated overnight to allow them to attach, then exposed to different treatments, e.g., DGLA׳s radical derivatives and PGs (0.1 to 10 μM), 5-FU (0.25 to 1.0 mM), and a combination (5-FU and a radical derivative). After 48 h incubation at 37 °C, 20 µL per well of CellTiter^®^ 96 Aqueous One Solution Reagent was added to each well. After additional 4 h incubation in the incubator, the quantity of formazan product, which is proportional to the number of living cells, was assessed by recording the absorbance at 490 nm with a 96-well plate reader (SpectraMax M5; Molecular Devices). Cell viability was calculated as a percentage of the control group (treated with vehicle).

### Cell cycle analysis (PI staining)

Cells were seeded at ~40% confluency and incubated overnight to allow them to attach. After being exposed to different treatments (DGLA׳s radical derivatives and PGs ~1.0 μM) for 48 h, cells were harvested, washed with PBS and fixed with 1 mL of cold 70% ethanol at 1×10^6^ cells/mL for 30 min at 4 °C. After centrifugation, the supernatant was discarded. The cells were washed with PBS, centrifuged and treated with 10 μL ribonuclease (10 mg/mL) at room temperature for 5 min. Then 400 μL of propidium iodide (PI, 50 μg/mL) was added to the sample. The cell cycle distribution was measured after a 30 min incubation using an Accuri C6 flow cytometer (Becton–Dickinson, NJ, USA), and 10,000 cells were counted for each sample. The percentage of cells in different phases of the cell cycle was analyzed by FlowJo (TreeStar, Ashland, OR, USA).

### Cell apoptosis assay (Annexin V-FITC/PI staining)

Cell apoptosis was assessed using an Annexin V Apoptosis Detection Kit I (BD Pharmingen™, NJ, USA) according to the manufacturer׳s instructions. Briefly, the cells were seeded overnight at ~40% confluency and exposed to different treatments, including radical derivatives (1.0 μM), 5-FU (0.5 mM), and a combination. Then the cells were trypsinized, washed with cold PBS and re-suspended in 1× binding buffer at a concentration of 1×10^6^ cells/mL. After 100 μL of the sample solution was transferred to a new tube, 5 μL of FITC Annexin V and 5 μL of PI solution were added. The cells were gently vortexed and incubated for 15 min at 25 °C in the dark. Then 400 μL of 1× binding buffer was added to each sample. The effect of different treatments on cell apoptosis was determined by an Accuri C6 flow cytometer within 1 h, and 10,000 cells were counted for each sample. Unstained cells and cells stained with FITC Annexin V only and PI only were used as controls to set up compensation and quadrants. Data was analyzed by FlowJo.

### Western blot

Cells were seeded, incubated overnight and exposed to different treatments. After 48 h of treatment, the protein was extracted and quantified. Equal amounts of protein extract (50 µg) were loaded into each well of 10% SDS-PAGE gels and run at a constant current of 30 mA for 1 h. The proteins were then transferred to nitrocellulose membranes at a constant voltage of 80 V for 2 h on ice. Membranes were blocked with 5% (w/v) non-fat milk in 1× Tris base saline solution with Tween-20 (TBS-T) for 15 min at room temperature. Then the membranes were incubated with primary antibodies (used at 1:600 dilution, Cell Signaling, MA, USA) overnight at 4 °C with continuous rocking. Membranes were washed 3 times for 5 min each with 1× TBS-T, then incubated with horseradish peroxidase (HRP)-conjugated secondary antibody (used at 1:2000 dilution, Cell Signaling, MA, USA) for 1 h at room temperature with continuous rocking. After washing 3 times with 1× TBS-T, the membranes were incubated in ECL Western blot substrates (Pierce, Thermo Fisher Scientific, UT, USA) for 1 min, and exposed to X-ray film (Phoenix Research Products, NC, USA). Luminescence signals were captured on a Mini-Medical Automatic Film Processor (Imageworks) with β-actin used as a loading control.

### Statistics

All data was assessed using an unpaired Student׳s *t*-test. A *p* value ≤0.05 was considered significant.

## Results

### Measurement of DGLA-derived radicals, PUFAs, and PGs from DGLA-treated colon cancer cells

LC/MS analysis was applied to quantify PUFAs, PGs and DGLA-derived free radicals (in reduced form, hydroxylamines) from 2×10^6^ HCA-7 colony 29 cells treated with 100 μM of DGLA for up to 48 h. Among the three DGLA׳s free radical metabolites tested, the POBN adduct of the 1-hexanol radical (the common product formed from both DGLA and AA) was the most abundant product (79.24±5.68 nM, Table 1) at 12 h of incubation. The two exclusive DGLA׳s free radicals, heptanoic acid radical and 8-hydroxyoctanoic acid radical, were much less abundant at up to 48 h at 7.28±0.79 and 8.49±0.39 nM, respectively.

Our data also showed that PGE1 (from COX-catalyzed DGLA peroxidation) was accumulated during the first 24 h of incubation, reaching ~146 nM before it started to decrease (Table 1). Meanwhile, a notable accumulation of PGE2 (from COX-catalyzed AA peroxidation) became dominant over the PGE1 and reached ~113 nM at 48 h. The effective accumulation of PGE2 from DGLA-treated cells indicated cellular D5D-mediated conversion of DGLA to AA. This conversion was confirmed by the measurement of much more AA (69.74±8.04) than DGLA (30.93±6.47 nM) in the cells treated with DGLA for 48 h. We also observed the formation of an exclusive AA radical (a carbon–carbon double bond-centered free radical), seen as the hydroxylamine of a POBN adduct with *m/z* 449, from HCA-7 cells after a 12 h incubation with DGLA [24]. The accumulation of AA as well as the exclusive AA radical at certain time points was also correlated with improved cell proliferation vs. the time points when no or limited AA and PGs-2 were accumulated (data not shown).

### DGLA׳s radical derivatives attenuate colon cancer cell proliferation

**Fig. 1:**
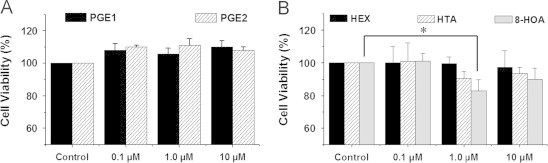
Comparison of cell growth responses of PGs vs. DGLA׳s radical derivatives. HCA-7 colony 29 cells were treated with A: PGs1 and PGs2, respectively, at 0.1 to 10 μM for 48 h; and B: DGLA׳s radical derivatives (HEX, HTA and 8-HOA) at 0.1–10 μM for 48 h. Cell proliferation was examined by the MTS assay. Cell viability was presented as the percentage compared to control (treated with vehicle). Data represented three separate experiments run in triplicate per condition. (^⁎^*p*<0.05).

As the major metabolites from COX-catalyzed PUFA peroxidation, prostaglandins have been reported to possess diverse bioactivities, including anti-inflammation and anti-cancer growth [5,11,12,15]. We thus directly tested the effects on colon cancer cell growth of a range of PGs concentrations (0.1 μM–10 μM). This concentration range was chosen based on the PG production profile from our cell experiments, e.g., ~0.1 μM PGs were measured at 48 h from cells treated with 100 μM DGLA (Table 1). Our experiments showed that neither PGE1 nor PGE2 at the tested concentrations influenced proliferation of HCA-7 colony 29 cells at 48 h (Fig. 1).

We further tested the effect of three of DGLA׳s free radical derivatives, HTA, 8-HOA and HEX, on colon cancer cell growth in the range of 0.1 μM to 10 μM (Table 1). Our results showed that the two exclusive DGLA׳s radical derivatives, 8-HOA and HTA, both influenced proliferation of HCA-7 colony 29 cells at ~1.0 μM; 8-HOA exerted the most significant anti-proliferative effect. However, HEX, the common product of AA and DGLA, did not influence cell proliferation at the tested concentrations (Fig. 1). This comparison experiment indicates that, at certain concentrations, it is the exclusive DGLA׳s free radical derivatives from COX-catalyzed lipid peroxidation, rather than PGs, that are actually responsible for DGLA׳s anti-cancer bioactivities.

### DGLA׳s radical derivatives arrest cell cycle progression

**Fig. 2:**
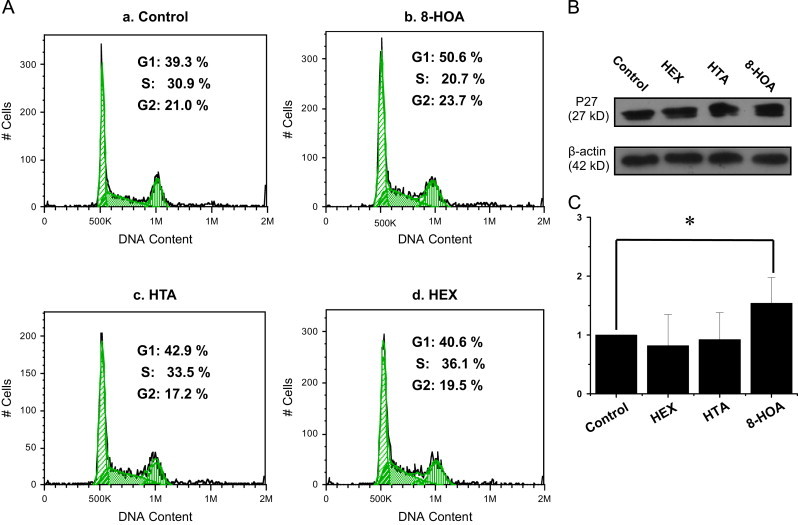
Effect of DGLA׳s radical derivatives on cell cycle distribution. A: Cell cycle distribution of HCA-7 colony 29 cells treated with DGLA׳s radical derivatives as well as PGs at 1.0 μM for 48 h; B: p27 expression in Western blot after 48 h of treatment with DGLA׳s radical derivatives (1.0 μM); and C: Quantification of p27 expression rate compared to control (treated with vehicle). The p27 expression rate in individual treatments was calculated as the ratio to beta-actin, and the rate in the control group was normalized to 1. (^⁎^*p*<0.05 from *n*≥3).

In order to understand how DGLA could regulate colon cancer cell growth, the HCA-7 colony 29 cells were treated with three of DGLA׳s free radical derivatives, HEX, HTA and 8-HOA, at 1.0 μM for 48 h. Cell cycle distribution analysis (via flow cytometer after PI staining) suggested that the exclusive DGLA׳s radical derivative (8-HOA) could induce G1 arrest (50.6% cells in G1) during the cell cycle progression compared to control (39.3% in G1). However, no significant changes were observed from the other two radical derivatives (42.9% and 40.6% cells in G1 phase, respectively, Fig. 2). Again, when the same amounts of PGs were used to treat cells, there were no differences in cell cycle distribution observed vs. control (data not shown).

To investigate the mechanism of the observed cell cycle arrest, we also tested various molecular targets that could be involved in cell cycle progression, including the cell cycle inhibitors p21 and p27, and cyclin D. Of these targets, p27 was found to be up-regulated by treatment with 8-HOA, which could be responsible for the observed cellular G1 arrest (Fig. 2).

### DGLA׳s radical derivatives induce cell apoptosis

**Fig. 3:**
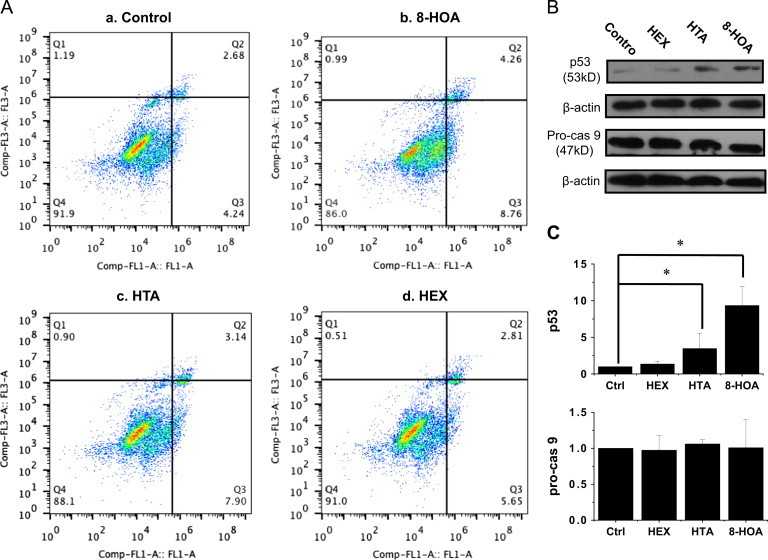
Effect of DGLA׳s radical derivatives on cell apoptosis. A: Cell apoptosis analysis of HCA-7 colony 29 cells treated with DGLA׳s radical derivatives at 1.0 μM for 48 h. Cell apoptosis was tested via FITC Annexin V/PI double staining; B: p53 and pro-caspase 9 expression in Western blot after 48 h of treatment with DGLA׳s radical derivatives (1.0 μM); and **C**: Quantification of p53 and pro-caspase 9 expression rate compared to control (treated with vehicle). The protein expression rate in individual treatments was calculated as the ratio to beta-actin, and the rate in the control group was normalized to 1. (^⁎^*p*<0.05 from *n*≥3).

We further assessed cell apoptosis via the FITC Annexin V/PI double staining method to determine whether DGLA׳s radical derivatives could induce cell death by promoting cell apoptosis. Consistent with the cell proliferation profile (Fig. 1), the two exclusive DGLA׳s radical derivatives, e.g., 8-HOA and HTA, promoted apoptosis of HCA-7 colony 29 cells. For example, we observed a significantly increased cell population in early (8.76%) and late (4.26%) apoptosis from the 8-HOA treatment compared to the control (4.24% and 2.68%, respectively, Fig. 3), while there was again almost no effect from the HEX treatment on apoptosis compared to the control.

In order to understand the mechanism of apoptosis in HCA-7 colony 29 cells induced by DGLA׳s radical derivatives, we also examined two key proteins that regulate the apoptotic pathway: the cancer suppressor p53 and the initiator caspase 9. Our data showed that the exclusive DGLA׳s radical derivatives HTA and 8-HOA significantly influenced p53 expression levels in cells, and the up-regulated p53 could be responsible for the promoted cell apoptosis. However, no significant changes were observed in pro-caspase 9 levels for any of the three DGLA׳s radical derivatives (Fig. 3).

### DGLA׳s radical derivatives enhance the cytotoxicity of 5-FU

**Fig. 4:**
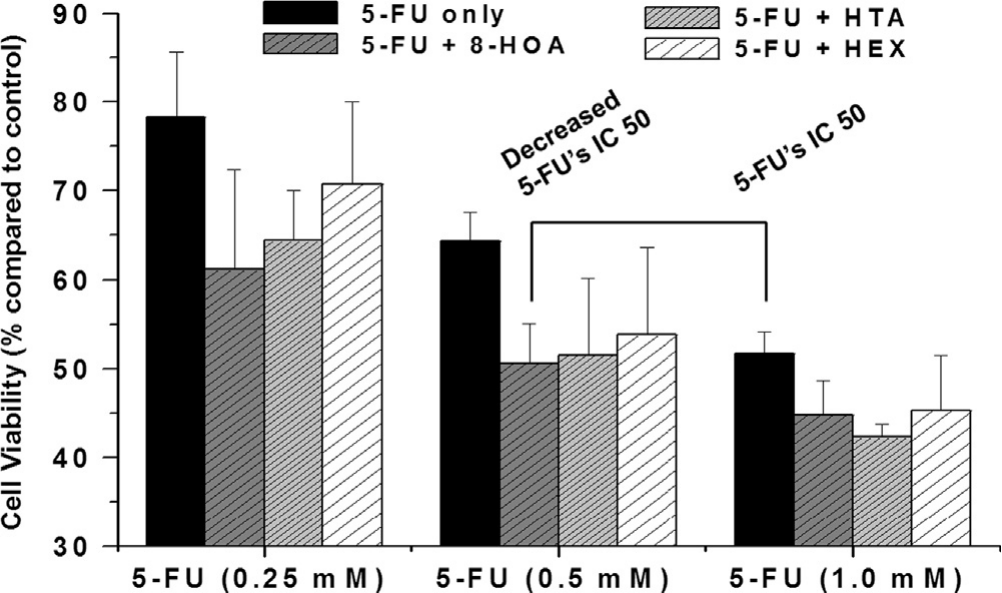
Cell growth response from combined treatment of 5-FU and DGLA׳s radical derivatives. HCA-7 colony 29 cells were treated with 5-FU (0.25, 0.5 and 1.0 mM) alone or 5-FU combined with DGLA׳s radical derivatives (1.0 μM) for 48 h. Cell proliferation was assessed by the MTS assay. Cell viability was presented as the percentage compared to control (treated with vehicle). Data represented three separate experiments done in triplicate per condition.

5-FU, a pyrimidine analog, is the most widely used chemo-drug for colon cancer therapy. It can be incorporated into RNA and DNA and interfere with nucleoside metabolism, thereby leading to cell death [35–40]. However, it is known that many cancer cell lines exhibit drug resistance toward 5-FU treatment [27–28,31]. The IC50 of 5-FU (~1.0 mM) for HCA-7 colony 29 cells was obtained from our experiment (Fig. 4) in agreement with other research [31]. We then asked whether DGLA׳s radical derivatives could be used to enhance the cytotoxicity of 5-FU to HCA-7 colony 29 cells. A decreased IC 50 (~0.5 mM) for 5-FU was achieved when HCA-7 colony 29 cells were co-treated with 5-FU and DGLA׳s radical derivatives (e.g. 1.0 μM 8-HOA). The ability to improve 5-FU׳s efficacy with the exclusive DGLA׳s radical derivatives, might represent a potential strategy for colon cancer treatment using a combination of this chemotherapy drug and dietary care. We also found that co-treatment with DGLA (100 μM) could further decrease 5-FU׳s IC50 (~0.2 mM) if D5D knockdown HCA-7 colony 29 cells were used (data not shown).

**Fig. 5:**
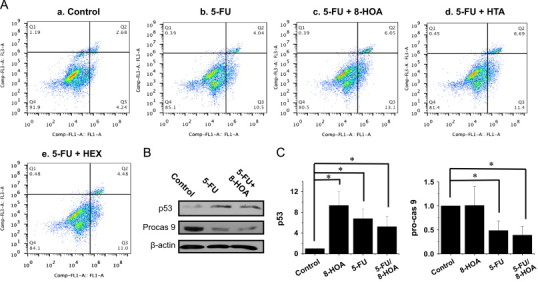
Effect on cell apoptosis from combined treatment of 5-FU and DGLA׳s radical derivatives. A: Cell apoptosis analysis of HCA-7 colony 29 cells treated with 5-FU (0.5 mM) alone or 5-FU combined with DGLA׳s radical derivatives (1.0 μM) for 48 h; B: p53 and pro-caspase 9 expression in Western blot upon different treatments for 48 h; and C: Quantification of p53 and pro-caspase 9 expression rate compared to control (treated with vehicle). The protein expression rate in individual treatments was calculated as the ratio to beta-actin, and the rate in the control group was normalized to 1. (^⁎^*p*<0.05 from *n*≥3).

Evidence suggests that 5-FU-induced cytotoxicity is also associated with apoptotic progress [37–40], similar to our observed anti-cancer effects from DGLA׳s radical derivatives. We observed that the combined treatment with 5-FU (0.5 mM) and 8-HOA (1.0 μM) induced cell apoptosis to a greater extent than 5-FU or 8-HOA treatment alone (Fig. 3), while combinations with HTA and HEX did not induce further apoptosis (Fig. 5). Further mechanistic studies also showed that not only did 5-FU and 8-HOA alone influence cellular p53 level, but that the combination also significantly up-regulated the level of p53 expression (Fig. 5). In addition, the observed activation of pro-caspase 9 with the combined treatment suggests that certain caspase pathways may be responsible for promoting cell apoptosis from the combination of 5-FU and 8-HOA.

## Discussion

Previous studies suggested that DGLA could produce two exclusive free radicals through a unique C-8 oxygenation during COX-catalyzed lipid peroxidation, and that the radical derivatives formed might be responsible for DGLA׳s anti-cancer activities [23–26]. In the present study, we have further investigated the effects and mechanism of DGLA׳s free radical derivatives on the colon cancer cell growth response, i.e., cell proliferation, cell cycle distribution, and apoptosis, with the HCA-7 colony 29 cell line. We also discovered that the exclusive DGLA׳s free radical derivatives can enhance 5-FU׳s cytotoxicity towards HCA-7 colony 29 cells. The outcome of this study might guide us to design a novel strategy combining the chemo-drug and dietary care in colon cancer treatment in the near future.

Quantification of PUFAs, PGs, and free radicals (spin trapped) showed that the amount of AA (and related metabolite PGE2) dominated over DGLA (and related metabolite PGE1) in HCA-7 colony 29 cells after 48 h of DGLA treatment (Table 1). The results indicated that cellular D5D efficiently converted DGLA to AA during our experiments. The conversion could restrict DGLA׳s anti-cancer bioactivity [24] and also corresponded to formation of the highly abundant hexanol radical (the common radical metabolite from DGLA and AA peroxidation) and two low abundance DGLA-derived radicals (the exclusive 8-HOA and HTA radicals). The observed different amounts of free radicals from C-8 and C-15 oxygenation in DGLA peroxidation may also be due to the different affinities of COX towards the two oxygenation mechanisms.

Depending on the product series, prostaglandins have been reported to possess diverse activities, including pro- and anti-cancer effects [5,11–12,15,17]. However, no effects of either PGE1 or 2 on cell proliferation were found under our experimental conditions (Fig. 1), probably due to our low but more physiologically relevant concentrations compared to the other research [15,17]. We believe that our concentrations were more appropriately selected to test the cellular growth response. The formation of free radicals during our experiments may be greatly underestimated by the cellular spin-trapping-based method. We are currently working on a GC/MS-based analytical method to better quantify DGLA׳s radical derivatives and assess their associated activity. Considering all these factors, we propose that the exclusive DGLA-derived radical metabolites from COX-catalyzed peroxidation are likely to be at least partially responsible for DGLA׳s anti-cancer bioactivity in physiological settings. We are also conducting an experiment to quantify the non-free-radical metabolites from COX-catalyzed DGLA peroxidation.

To investigate how DGLA׳s radical derivatives regulate colon cancer cell growth, we examined the cell cycle distribution and apoptosis. Our studies showed that the exclusive DGLA׳s radical derivative (8-HOA) induces cell cycle arrest at the G1 phase (Fig. 2), probably due to the induction of p27, a cyclin-dependent kinase inhibitor. Another cell cycle inhibitor, p21, is also commonly involved in cell cycle arrest at the G1/S phase and tightly controlled by p53 [41,42]. We observed a significantly up-regulated expression of p53 upon treatment with DGLA׳s radical derivatives (Fig. 3). However, because the expression level of p21 in the experiments was too low, we were unable to determine whether the p53-dependent p21 pathway is also involved in arresting HCA-7 colony 29 cells in G1. We are now also conducting a mechanistic study on the expression of these genes in order to understand the effect of DGLA׳s radical derivative, and therefore DGLA peroxidation via COX, on cell cycle progression.

Our apoptosis studies showed that the exclusive DGLA׳s radical derivatives could induce cell apoptosis, most likely mediated by the induction of p53, a cancer suppressor gene (Fig. 3). It has been well documented that reactive lipid species from lipid peroxidation can directly modify mitochondrial proteins and lead to cytochrome c release [43,44]. Thus, we propose that the exclusive DGLA׳s radical derivatives can interfere with mitochondrial proteins and lead to cell apoptosis through mitochondrial pathways. Pro-caspase 9, another mediator for cell apoptotic events, can be cleaved into an active form via either p53-mediated or p53-independent mechanisms, i.e., directly triggered by cytochrome c release from mitochondria. However, no significant changes were observed in expression of pro-caspase 9 in cells from DGLA׳s radical derivative treatments (Fig. 3).

5-FU is the most widely used chemo-drug for cancer therapy, especially for colon cancer. However, the major limitation in applying 5-FU in cancer therapy is its chemo-resistance. Evidence shows that co-treatment with indomethacin and NS-398, both of which are COX inhibitors, could significantly sensitize colon cancer cells to 5-FU, and that the regulation of COX-mediated PUFA metabolism may be an effective strategy for overcoming 5-FU chemo-resistance [31]. Here we have also tested the ability of DGLA׳s radical derivatives to enhance 5-FU׳s cytotoxicity to HCA-7 colony 29 cells. A significantly improved 5-FU IC50 was obtained when the cells were treated with 5-FU combined with 8-HOA (Fig. 4). Our mechanism study has shown that the enhanced cytotoxicity from the combination treatment may be due to the enhanced induction of cell apoptosis triggered by an activation of pro-caspase 9 (Fig. 5). Interestingly, 8-HOA alone did not influence pro-caspase 9, while the combination of 8-HOA and 5-FU did induce the activation of pro-caspase 9, probably independent of p53 (Fig. 5).

COX is overexpressed in >80% of adenocarcinomas and has a substantial association with cancer development [18–20]. COX inhibition has become a conventional strategy for the treatment of cancers, by virtue of its inhibition of the over-production of PGs-2 (metabolites of COX/AA peroxidation). However, the results from our series of work show that, under the catalysis of COX, DGLA can produce beneficial free radical metabolites associated with anti-cancer activity. This new observation inspires us to take advantage of the high COX expression in cancer cells to control cancer cell growth by eliciting DGLA peroxidation to form exclusive DGLA׳s radicals. In sum, the present study provides the first evidence demonstrating that the exclusive DGLA׳s free radical derivatives could lead to cell growth inhibition, cell cycle arrest and apoptosis in the HCA-7 colony 29 cell line from up-regulating the cancer suppressor p53 and the cell cycle inhibitor p27. We also found that the exclusive DGLA׳s free radical derivatives could significantly improve the cytotoxicity of 5-FU to cells insensitive to it. The enhanced cytotoxicity observed from the combination treatment may be due to enhanced induction of apoptosis triggered by activation of pro-caspase 9. The outcome of this work may lead us to the development of a novel drug combination strategy for colon cancer therapy.

## Figures and Tables

**Table 1:** Profile of PUFAs, PGs, and free radicals (trapped by POBN) from HCA-7 cells treated with DGLA (100 μM). PUFAs and PGs from 2×10^6^ cells treated with DGLA (100 μM) were measured via LC/MS analysis using internal standards, including AA-d_8_, DGLA-d_6_, PGE1-d_4_ and PGE2-d_9_. The formation of free radicals (trapped by POBN) from 2×10^6^ cells treated with POBN (20 mM) and DGLA (100 μM) was quantified by LC/MS analysis. Deuterated-POBN (d_9_-POBN) was used as an internal standard for hydroxylamine quantification [22–24,27]. Data are represented as mean±SD, *n*=3.

Time (h)	[PUFAs] (nM)		[PGs] (nM)		[DGLA free radicals] (nM) trapped by POBN as hydroxylamine		
	DGLA	AA	PGE1	PGE2	HEX	HTA	8-HOA
12	562.6±9.556	301.0±55.77	86.57±13.55	85.13±9.277	79.24±5.685	7.282±0.793	7.142±0.866
24	541.4±7.938	304.0±60.29	145.9±8.712	104.5±26.58	65.69±4.531	5.800±1.10	8.488±0.386
48	30.93±6.474	69.74±8.040	71.36±2.627	113.2±21.26	59.47±3.850	4.654±0.693	6.978±0.750
